# The Wastrels of Modern Cities

**Published:** 1896-01-18

**Authors:** 


### Modern Sociology.

### THE WASTRELS OF MODERN CITIES.

The operation of sorting or sifting any heterogeneous mass of material has one inevitable result. When all the useful elements have been classified and respectively applied to their proper purposes a residuum remains of no appreciable value and no perceptible use—broken fragments, maybe, of things once well thought of and fair to look upon—possibly the mere dust and ashes thereof, or elements altogether foreign to their surroundings; a conglomeration of rubbish to be incontinently scattered to the winds as dirt, defiling all it comes in contact with. Communities of human beings are always undergoing this process of sorting and sifting with much the same ultimate result. To a large extent the operation is a spontaneous one; talent and industry bearing some individuals to the very surface of the social firmament, supporting others with a less degree of buoyancy at lower levels; whilst others again, lacking these good qualities, and depressed by the dull weight of laziness and incapacity, grope helplessly in the mire of the lowest depths of all. Broken and battered by rough usage they quickly cast off all such redeeming qualities as they may ever have possessed. They shrink from the knowledge of their fellows as from the light of day, and seek to hide their degraded identity in the dark corners of our great cities. They are the wastrels of society; the children unhappily born to them inherit the taint of idleness and evil, and generally grow up wastrels too. They neither toil nor spin, honestly or dishonestly, for their very vices are practised in a desultory manner, and they exist merely as a problem for the social reformer and the philanthropist to solve. How? The sifting process has for years been in full swing among them. Here and there one has been rescued—some, perhaps, are being helped upwards now; but the residuum remains. How shall we deal with it? We cannot scatter it to the winds, like the detritus of inanimate matter, or apply to it any drastic process of extermination. We are painfully conscious of its defiling presence; it frets us even as cobwebs fret the soul of the careful housewife; but we cannot, like her, apply the housemaid's broom and sweep the evil thing away. Yet, as a law-abiding race of cleanly morality, we feel that the taint on our social reputation must be removed, or, at least, kept within the narrowest possible limits; and it is well that we should occasionally rouse ourselves to a serious contemplation of the unsavoury subject. Not in any unsympathetic spirit, however, for the rights and privileges of fellow-creatures are involved, and no remedy for the evil can possibly be effectual which is not based upon the strictest principles of honesty and justice, and the divine law of love. It is but accident that we ourselves are the commentators instead of the subjects of comment; and bearing this in mind we shall be the better able to appreciate the cruelty of our existing laws, which make it a crime for a man who never owned a bed of his own to wander abroad and sleep in the open air. The futility of such a remedy is established by its own miserable failure. And it is abundantly clear that in this, as in other social questions where the rights of humanity are concerned, *force majeure*, whilst it may scourge, is of no avail to set things right. The task must be undertaken in a widely different spirit. The old law of suppression must be abrogated. First causes must be sought out and dealt with, not as crimes, but as moral diseases. Indeed, it is a pathological question of some interest whether the unhappy creatures under consideration are not in a degree mentally defective. Certainly it seems strange that any human beings in full possession of their faculties should deliberately choose lives of abject misery, and be incapable of any effort to extricate themselves and rise to better things. Be this as it may, it is clear that help is needed; enforced help, kindly administered, but with the force of the law behind it, so that it may not fail of reaching its utmost aim. By such or similar means we have cleared the streets of our towns and villages of the wandering idiots and lunatics who infested them but a few years ago. These poor creatures are no longer permitted the liberty to air their delusions and eccentricities in public, to become the scoff of the idle and the reproaches of all. The seclusion which is forced upon them is the truest kindness, and has had results far exceeding all anticipation. The wastrels, who, as a body, are but one remove from this unhappy class, might well be similarly dealt with. Under legally enforced detention in suitable institutions they might be redeemed from themselves, and transformed in time into comparatively useful members of society. Having learned some ordinary trade or practical industry they would be fitted for voluntary emigration to some of those vast colonies of ours across the seas, where their presence would be more than welcome; and where opportunities, impossible in this country, would lie open before them. And even those who, by reason of age or bodily infirmity, were unfitted for emigration, or who preferred to remain here, would be removed by their training from their original condition of abject misery, and would at least have acquired some habits of industry and the means of earning a decent livelihood. The substitution of some such treatment as this for the harshness of prison discipline or the indolence of the workhouse would be distinctly advantageous in every way, and would well repay the time and attention of the Legislature.

